# Unveiling the peripheral nerve hallmarks of chemotherapy-induced neuropathy: insights from paclitaxel treatment in a murine model

**DOI:** 10.1016/j.ynpai.2025.100200

**Published:** 2025-10-08

**Authors:** Maria Maiarù, Andrea Petrini, Federica De Angelis, Francesca Nazio, Sara Marinelli

**Affiliations:** a Department of Pharmacology, School of Pharmacy, University of Reading, Reading RG6 6UB, United Kingdom; b Sapienza University of Rome, Faculty of Mathematical, Physical and Natural Sciences, Department of Biology and Biotechnology “Charles Darwin” Neurobiology Degree, 00185 Rome, Italy; c National Council of Research, Institute of Biochemistry and Cell Biology (IBBC), 00015 Monterotondo (RM), Italy; d Department of Biology, University of Rome Tor Vergata, 00133 Rome, Italy

**Keywords:** Neuropathic pain, Neurotoxicity, Metabolic alterations, Schwann cells, Inflammation, Myelin degeneration, Innate immunity

## Abstract

Chemotherapy-induced peripheral neuropathy (CIPN) is a frequent and debilitating side effect of anticancer drugs like paclitaxel, significantly reducing the quality of life for cancer patients. Paclitaxel-induced peripheral neuropathy (PIPN) is primarily characterized by sensory disturbances such as mechanical allodynia and thermal hyperalgesia. Despite its prevalence, the mechanisms driving PIPN are not fully understood, and current treatment options remain limited. This study explores the impact of varying doses of paclitaxel on neuropathic pain, nerve structural changes, and metabolic alterations in a mouse model. Behavioural assessments demonstrated that paclitaxel induced dose-dependent mechanical allodynia and thermal hyperalgesia, with prolonged symptoms at higher doses. Furthermore, sciatic nerve dysfunction was observed, while metabolic tests revealed significant disruptions in glucose and triglyceride levels, suggesting a link between metabolic imbalances and neuropathy. Histological and molecular analyses identified increased TRPV1 and CGRP expression in skin nerve fibers, accompanied by Schwann cell dysfunction, characterized by myelin disorganization, decreased levels of myelin proteins (P0, MBP), and elevated LC3 levels, pointing to autophagy involvement. Moreover, infiltration of macrophages and mast cells into sciatic nerves indicated an innate immune response. These results emphasize the complex nature of PIPN, which involves sensory nerve sensitization, Schwann cell damage, and metabolic dysregulation. Elucidating these pathways could inform the development of more effective therapies aimed at preventing or alleviating the impact of CIPN.

## Highlights

• Paclitaxel induces dose-specific neuropathic pain symptoms and sciatic nerve dysfunction.
• TRPV1 and CGRP expression in skin nerve endings is significantly upregulated.
• Schwann cell dysfunction is linked to disrupted myelin and altered autophagic mechanisms.
• Metabolic changes in glucose and triglycerides correlate with neuropathy development.
• Macrophage and mast cell infiltration suggests an inflammatory response in neuropathy.

## Graphical abstract

**Figure:**
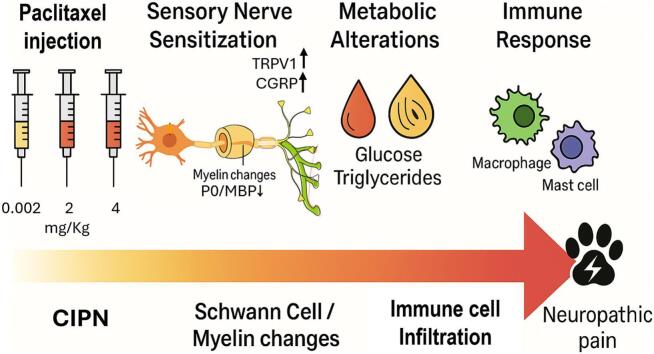

Cancer, the second leading cause of death worldwide, is expected to see 28.4 million new cases by 2040 (Sung et al., 2021). Key chemotherapeutic drugs, such as platinum-based antineoplastics, taxanes, vinca alkaloids, proteasome inhibitors, and immunomodulatory drugs, are crucial for treatment (Zajączkowska et al., 2019, Starobova and Vetter, 2017). However, these drugs also harm healthy cells, causing side effects such as chemotherapy-induced peripheral neuropathy (CIPN), characterized by spontaneous pain (pain occurring without external stimuli), paraesthesia (hypersensitivity), and allodynia (pain provoked by innocuous stimuli), often beginning in the fingers, feet, and hands, and sometimes progressing toward the body (Zajączkowska et al., 2019, Starobova and Vetter, 2017, Colvin, 2019). Some patients also experience motor dysfunctions and rare autonomic symptoms (Zajączkowska et al., 2019, Starobova and Vetter, 2017).

The severity of CIPN symptoms varies based on the drug, dosage, and treatment timing, indicating differences in neurotoxicity mechanisms (Colvin, 2019). For example, paclitaxel- and oxaliplatin-induced neuropathy can present shortly after treatment starts, resembling acute peripheral neuropathy, with symptoms sometimes fading after chemotherapy ends (Zajączkowska et al., 2019, Colvin, 2019). Conversely, chronic peripheral neuropathy can develop later, persisting long after treatment, a phenomenon known as “coasting” (Zajączkowska et al., 2019, Starobova and Vetter, 2017, Colvin, 2019). The risk of chronic CIPN increases with higher cumulative doses and longer exposure to the drug (Colvin, 2019).

Paclitaxel-induced peripheral neuropathy (PIPN) primarily presents sensory symptoms such as mechanical allodynia/hyperalgesia, tingling, and numbness (Taguchi, 2016). These symptoms significantly affect patients' quality of life, causing burning pain, cold and mechanical allodynia, anxiety, and cognitive loss (Klein and Lehmann, 2021, Paltian et al., 2022). Although PIPN severely impacts cancer patients and survivors, the exact mechanisms remain unclear, and as a consequence, effective treatments are lacking.

It has been shown that paclitaxel disrupts calcium signalling, damages mitochondria, induces reactive oxygen species formation, and affects neuropeptide and growth factor release in sensory neuron axons, as well as immune and glial cells (Staff et al., 2020). It activates ion channels responsive to extracellular cues and involves matrix-metalloproteinase 13 (MMP-13) in neuropathy (Staff et al., 2020). Paclitaxel cannot cross the blood–brain barrier (Alqahtani et al., 2019) and directly affects the peripheral nervous system (PNS). Various mechanisms, though not fully understood, have been identified. Disruptions in the cytoskeleton and axon morphology are implicated, with differential gene expression patterns noted in breast cancer survivors with PIPN (Kober et al., 2019). Paclitaxel binds to and stabilizes microtubules, disrupting their organization and impairing axonal transport (Bober et al., 2015). Additionally, changes in microtubule organization and mechanical loading of neurons exacerbate transport deficits (Bober et al., 2015).

Current understanding of paclitaxel's effects on myelin structure and Schwann cell (SC) alterations in peripheral neuropathy is inconsistent. Paclitaxel induces SC dedifferentiation, increasing p75 and galectin-3 expression, leading to demyelination, a key factor in chemotherapy-induced peripheral neuropathy (CIPN) (Koyanagi et al., 2021). However, electron microscopy and X-ray diffraction (XRD) studies show no significant impact on internodal myelin structure (Gilardini et al., 2012). Research on gene expression changes in paclitaxel-induced neuropathy indicates involvement of both degenerative and regenerative processes, with minimal immune response and notable sex-dependency (Naratadam et al., 2024). Comparative studies in rats reveal that both paclitaxel and docetaxel reduce nerve conduction velocity, with paclitaxel having more severe effects on nerve fibers (Rattanakrong et al., 2022).

These findings underscore the complex and multifaceted impact of paclitaxel on peripheral nerve health, involving both functional and structural changes. To better understand these effects, we investigated various paclitaxel doses in a murine model, focusing on neuropathic pain, functional alterations, and metabolic, structural, immune, and molecular changes in the peripheral nervous system, including skin receptors, nerve endings, and nerves. Metabolic changes were also assessed to determine if the preclinical mouse model replicates the clinical condition, as previous studies have reported metabolic alterations in humans and rats following paclitaxel treatment (Panis et al., 2017, Micheletti et al., xxxx).

Based on these findings, this work aims to provide an overview of the phenomena involving peripheral nerves in paclitaxel toxicity, guiding future research towards identifying new therapeutic targets for developing effective preventive or curative treatments.

## Methods

### Experimental design

**Fig. 1:**
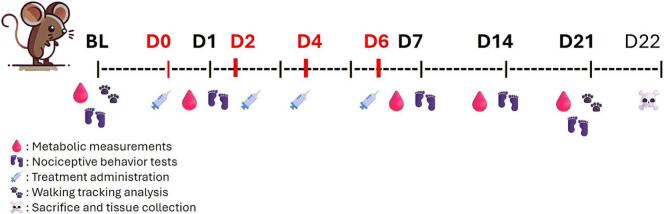
Timeline of the experiments. BL = Baseline, in red the days of treatment starting from day 0 (D0), in bold the days of tests. Sacrifices and tissues collection at the end of experiments (day 22 – D22). (For interpretation of the references to colour in this figure legend, the reader is referred to the web version of this article.)

The animals were randomly assigned to three groups: two experimental groups receiving paclitaxel at doses of 0.002 mg/kg (n = 9), 2 mg/kg (n = 8), or 4 mg/kg (n = 8), and a control group receiving a vehicle solution (n = 11). To evaluate the development and maintenance of neuropathic symptoms, behavioural tests assessing somatosensory and motor functions were conducted Fig. 1. Additionally, metabolic measurements were taken to assess metabolic effects of paclitaxel treatment and potential correlations with symptom progression. These tests were performed before treatment to establish a baseline (BL), the day after treatment initiation (D1) to determine effects of a single injection, the day after the final injection (D7) to assess metabolic and somatosensory changes, and weekly thereafter (D14 and D21) to monitor symptom progression over time. Animals treated with 4 mg/kg of paclitaxel also underwent walking tracking analysis at baseline (BL), end of treatment (D8), and weekly thereafter (D15 and D22). Following the final testing day (D22), animals were sacrificed, and sciatic nerves and hind paw skin were collected for ex vivo molecular and immunohistochemical analysis of paclitaxel-induced alterations.

### Drugs

Paclitaxel (Sigma-Aldrich) was dissolved in a stock solution made up of 50% absolute ethanol and 50% Cremophor (Sigma-Aldrich) and stored at −20°C for a maximum of 14 days. On the day of the injection, stock solution was diluted in 0.9% saline for a final mixture of 1 part of ethanol: 1 part of Cremophor: 58 parts of saline. 10 ml/kg of body weight were administered intraperitoneally (i.p.) for a final dose of 0.002, 2 or 4 mg/kg of paclitaxel. To mimic the human chemotherapeutic treatment, the injections were performed every other day for a total of 4 injections (days 0, 2, 4 and 6). On the same days, control animals received an equal volume of the vehicle solution.

### Animals

In all experiments, 4-month-old male C57BL6/J mice (EMMA Infrafrontier, Monterotondo, Rome – Italy) were used. Upon arrival at the laboratory (at least 2 weeks before the experiments), the animals were housed in standard transparent plastic cages lined with sawdust, under a standard 12/12-hour light/dark cycle (7:00 am/7:00 pm). Food and water were provided ad libitum. Animals were randomly assigned to either the treatment or vehicle group. All efforts were made to minimize animal suffering in accordance with Italian National Law (DL116/92, application of the European Communities Council Directive 86/609/EEC – Ministry of Health authorization nr. PR122/2019) on laboratory animal welfare and the guidelines of the Committee for Research and Ethical Issues of IASP (Zimmermann, 1983). The total number of animals used was minimized to the essential number needed to obtain statistically reliable results, as determined by a priori power analysis described in the statistics section.

## Behavioural tests

### Dynamic plantar aesthesiometer test

Mechanical allodynia was assessed using a dynamic plantar aesthesiometer (Ugo Basile, Gemonio, Italy) to measure the sensitivity of both hind paws to typically non-painful mechanical stimuli. Testing was conducted at baseline (BL) and on days 1, 7, 14, and 21 (D1, D7, D14, D21) following the first injection. Prior to BL testing, animals underwent a habituation session to reduce stress. Each testing day, animals acclimated to the experimental room for 30 min, then spent 10 min in plastic cages with wire mesh floors before testing. During the test, a movable force actuator was placed under the plantar surface of the hind paw, and a flexible metallic filament applied increasing force until the animal withdrew its paw. The force intensity (measured in grams) that triggered a withdrawal response was recorded. For each animal, the final withdrawal threshold was determined as the average of three measurements: one from the right hind paw, one from the left hind paw, and one randomly from either paw.

### Plantar test

Thermal hyperalgesia was evaluated by measuring the sensitivity of both hind paws to hot stimuli using a plantar test apparatus (Ugo Basile, Gemonio, Italy). Tests were conducted before the treatment began (baseline, BL) and on days 1, 7, 14, and 21 (D1, D7, D14, D21) following the first injection, always one hour after the dynamic plantar aesthesiometer test. Animals were placed on an elevated glass floor, and an infrared light source was positioned under the plantar surface of the hind paw. The time latency from the start of the hot stimulus to the withdrawal response was recorded. For each animal, the final time latency was calculated as the average of two measurements, one for each hind paw.

### Walking track analysis (sciatic static index)

To evaluate the functional effects of the treatment, walking track analyses were performed before the start of the treatment (baseline, BL) and on day 21 after the first injection. Before baseline testing, the animals underwent a habituation session. On each testing day, the animals were acclimated to the experimental room for 30 min. Footprints were obtained by dipping both hind paws in black ink and allowing the animals to walk freely along a corridor lined with white paper. The Sciatic Static Index (SSI) was calculated for each testing day to measure sciatic nerve function, using the following formula initially developed by Baptista and colleagues (19):

SSI = 101.3 × [(TS-BTS)/BTS] − 54.03 × [(PL-BPL)/BPL] − 9.5

where TS (toe spread) is the distance between the 1st and 5th toes, and PL (paw length) is the distance between the tip of the third toe and the most posterior aspect of the paw. BTS and BPL are the TS and PL obtained during baseline testing. For each animal, these parameters were measured on five footprints (2–3 footprints per paw) and averaged. The footprints were scanned and digitalized and measured with Image J tool straight and “measure.”.

### In vivo metabolic measurements

To assess the treatment's effects on metabolism, levels of blood glucose and triglycerides were measured using the MultiCare test-strip kit (Biochemical Systems International, Arezzo, Italy). To minimize variability due to differing feeding schedules among subjects, measurements were conducted after a 2-hour fast. Blood samples for all analyses were collected from capillary blood by tail clipping, performed before treatment (BL) and on days 1, 7, 14, and 21 (D1, D7, D14, D21) following the initial injection.

### Tissue collection

Twenty-two days after the first injection of paclitaxel or vehicle solution, the animals were anesthetized and sacrificed. The skin from each hind paw was collected and immersed in 4 % paraformaldehyde in phosphate-buffered saline (PBS) for immunohistochemistry. Next, sciatic nerves were extracted through a thigh incision and either immediately frozen in liquid nitrogen for Western blotting or immersed in 4 % paraformaldehyde in PBS for immunohistochemistry.

### Immunohistochemistry (IHC)

Tissues collected for immunohistochemistry were immersed in 4 % paraformaldehyde in PBS for 48 h. After 24 h in 30 % sucrose in PBS for cryoprotection, they were stored at −80 °C. Sciatic nerves and skin were embedded in OCT (VWR Chemicals) and sectioned into 20 μm slices using a cryostat (Leica Microsystem, model CM1850 UV). The sections were then mounted on glass slides and incubated with a 1:200 solution of primary antibody (Table 1) in 0.3 % Triton X-100 in PBS for 24 h at room temperature in a humidity chamber. After washing with PBS, they were incubated with a 1:200 solution of fluorophore-conjugated secondary antibody (Table 2) in 0.3 % Triton X-100 in PBS for 2 h at room temperature in a humidity chamber. The sections were washed again with PBS and counterstained by incubating them in a 1:1000 solution of Hoescht (Sigma-Aldrich) in 0.3 % Triton X-100 in PBS for 5 min at room temperature in a humidity chamber. Finally, they were washed with PBS and mounted using a mixture of 3 parts glycerol and 1 part PBS.

**Table 1:** List of primary antibodies for IHC.

Antibody	Host species	Supplier	Target
anti-CD11b	rat	Bio-Rad	Macrophages
anti-CGRP	rabbit	Calbiochem	Calcitonin gene related peptide fibers
Anti- Casp3	rabbit	Cell Signaling	Caspase 3 − apoptosis
Anti-TRPV1	Guinea pig	Millipore	Vanilloid receptors 1
anti-chymase	mouse	Santa Cruz	Mast cells
anti-GFAP	mouse	Sigma-Aldrich	Schwann cells
anti-LC3	rabbit	Sigma-Aldrich	Autophagy
anti-MBP	rabbit	Sigma-Aldrich	Myelin Basic Protein
anti-P0	chicken	Millipore	Myelin protein zero

**Table 2:** List of secondary antibodies for IHC.

Antibody	Supplier	Fluorophore
Donkey anti-chicken	Jackson ImmunoResearch	Rhodamine (red)
donkey anti-rabbit	Jackson ImmunoResearch	Alexa Fluor 647 (far red)
donkey anti-mouse	Jackson ImmunoResearch	Alexa Fluor 488 (green)
donkey anti-rat	Jackson ImmunoResearch	Rhodamine (red)
goat anti-guinea pig	Jackson ImmunoResearch	Rhodamine (red)
goat anti-mouse	Jackson ImmunoResearch	Rhodamine (red)

### Image analysis

Images were captured using a TCS SP5 confocal microscope (Leica Microsystems) through laser scanning confocal microscopy. To prevent channel cross-bleeding, all images were acquired in sequential scanning mode. The images were analyzed with ImageJ software (version 1.53; National Institutes of Health, Bethesda, MD, USA). Fluorescence of CGRP and TRPV1 in the skin and LC3 in the nerve was quantified using the RGB method, which converts pixels in brightness value for the specific channel (red, green, or blue). Total cell counts were automatically determined using ImageJ's tool for counting nuclei per image. For macrophage and mast cell counts, chymase- and CD11b-positive cells were manually counted using the counter and mark tool. Each mouse's data (N = 3–5 per group) were averaged from three different images for all analyses.

### Western blot (WB)

Sciatic nerves from vehicle-treated and paclitaxel-treated animals were homogenized, and the protein concentration of each lysate was determined using the Bradford assay. Standard samples with known concentrations of bovine serum albumin (BSA, Sigma-Aldrich) were dyed with Coomassie Brilliant Blue, which binds to aromatic residues of proteins. Their absorbance at 595 nm was measured using a spectrophotometer. A standard reference curve was created by plotting these absorbance values (y-axis) against the sample concentrations (x-axis). The unknown tissue lysates underwent the same procedure, and their protein concentrations were determined by comparing their absorbance values to the standard curve.

The samples were then diluted 3:1 in a buffer solution composed of 250 mM Tris-HCl (pH 6.8), 40 % glycerol, 20 % β-mercaptoethanol, and 0.002 % bromophenol blue. They were denatured at 100 °C for 5 min and immediately placed on ice. The samples, with equal protein concentrations (10 μg for myelin proteins and 70 μg for autophagic and apoptotic markers), were loaded into the wells of a polyacrylamide gel. The gel was immersed in a running buffer (0.025 M Tris-HCl, pH 8.3; 0.192 M glycine; 0.1 % sodium dodecyl sulfate) and subjected to a 30-mA current. The separated proteins were transferred onto a nitrocellulose membrane using a transfer buffer (25 mM Tris-HCl, pH 8.3; 5.5 % glycine; 20 % methanol) with a 70 V voltage for 2 h at 4 °C.

After blotting, the membrane was incubated for 1 h at room temperature in a blocking solution (5 % BSA in PBS or 5 % dry milk powder, depending on the antibody). The primary antibody (Table 3) was diluted in the blocking solution and incubated overnight at 4 °C, followed by washing with T-TBS (50 mM Tris-HCl, pH 7.5; 150 mM NaCl; 0.05 % Tween20). The secondary antibody, conjugated with peroxidase (Jackson ImmunoResearch), was diluted 1:20000 in 5 % dry milk powder in T-TBS, incubated for 1 h at room temperature, and then washed with T-TBS. Detection of the primary-secondary antibody complexes was performed using the ECL (Enhanced Chemiluminescence) method, and images were captured with a UVITEC instrument (Cambridge).

**Table 3:** List of primary antibodies for WB.

Antibody	Host species	Supplier	Dilution
anti-β Actin	mouse	Abgent	1:2000
anti-Casp 3	rabbit	Cell Signaling	1:600
anti-LC3	rabbit	Sigma-Aldrich	1:250
anti-MBP	mouse	Abcam	1:500
anti-P0	rabbit	Sigma-Aldrich	1:250

### Data analysis

Sample sizes for behavioral and metabolic tests were calculated beforehand using G*Power software (version 3.1.9.4) for repeated measure ANOVA (5 measurements, 4 groups) with a medium effect size (0.3), estimating a total of 32 subjects (approximately 8 per group). For immunohistochemical and molecular analyses, sample sizes were estimated based on previous experience and in line with the three Rs principle to minimize animal use.

For all experiments, data are presented as group means ± standard error of the mean (SEM). Behavioral and metabolic data were analyzed using repeated-measures analysis of variance (ANOVA), followed by post-hoc Tukey-Kramer tests. For groups with n < 5, non-parametric statistical analyses were used: immunohistochemical data were analyzed with the Kruskal-Wallis test (3 groups, n < 5), followed by post-hoc Bonferroni-Dunn tests. Western blot data were analyzed using unpaired Student’s t-tests. The significance level for all analyses was set at 0.05 (*p < 0.05; **p < 0.005; ***p < 0.001). Statistical analyses were performed using StatView software (version 5.0; Cary, NC, USA). Graphs were created with AlphaPlot software (version 1.011; https://alphaplot.sourceforge.io/).

In addition to reporting p-values, we calculated effect sizes (Cohen’s d) and 95 % confidence intervals (CI) for statistically significant comparisons. This approach allows to estimate the magnitude and biological relevance of observed differences, beyond binary significance testing. Effect sizes were computed for key behavioural and metabolic outcomes and are presented in Supplementary Table S1.

All statistical analyses are detailed in the figure legends.

## Results

### Impact of paclitaxel doses on nociceptive behaviour

Given that neuropathy development after paclitaxel treatment appears to be dose-dependent, we used three different doses: two categorized as ultralow (0.002 mg/kg) and low (2 mg/kg), and one considered the standard dose (4 mg/kg) for inducing neuropathy in mice (20). The ultralow dose was intentionally included with the aim of identifying a potentially ineffective concentration, in order to explore the threshold of paclitaxel-induced responses.

**Fig. 2:**
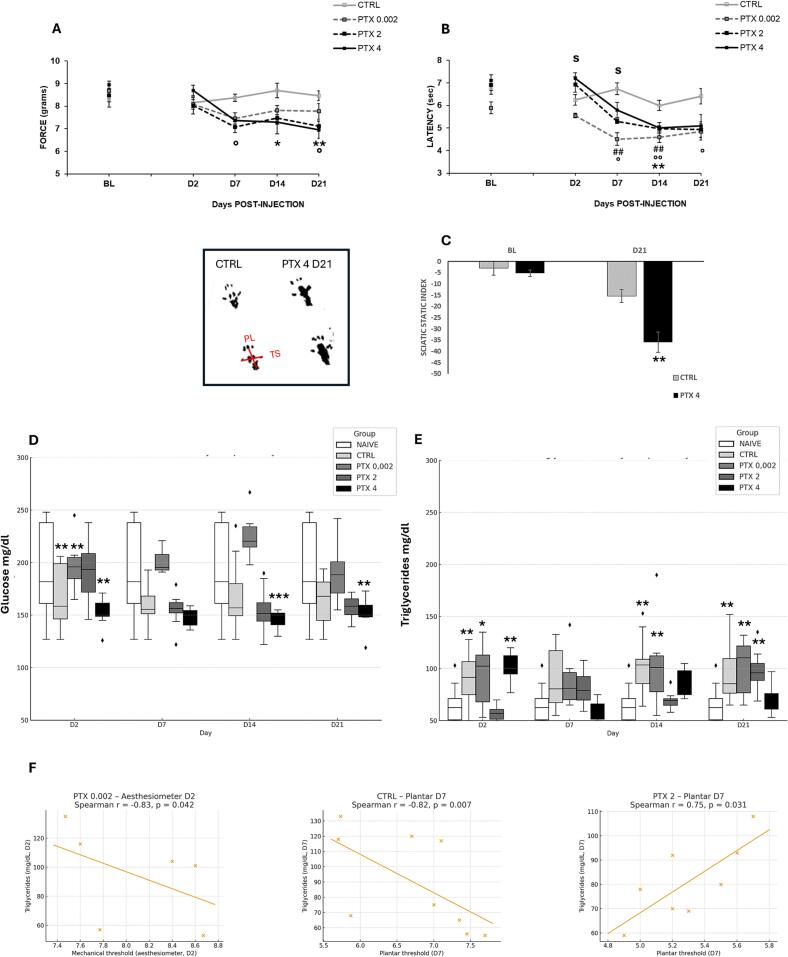
Nociceptive behaviour and metabolic alterations following paclitaxel administration. (A) Mechanical threshold (allodynia development) in baseline (BL) condition and at day 2 (D2), D7, D14 and D21 of vehicle (CTRL) or Paclitaxel (PTX) − treated mice at the dose of 0.002, 2 and 4 mg/kg, administered 4 times on alternate days (D1, D3, D5, D7). A two-way ANOVA for repeated measures revealed a significant effect for treatment (F_3,26_ = 4,48; p = 0,0089), time (F_4,12_ = 10,439; p < 0,0001) and treatment × time (F_12,104_ = 2,826; p = 0,002); Tukey-Kramer post hoc test: PTX 2 (°) P < 0.05; PTX 4 (*) P < 0.05; (**) P < 0.005 vs CTRL (CTRL N = 9; PTX 0.002 N = 6; PTX 2 N = 8; PTX 4 N = 7). (B) Thermal threshold (hyperalgesia development) in baseline (BL) condition and at day 2 (D2), D7, D14 and D21 of vehicle (CTRL) or Paclitaxel (PTX) − treated mice at the dose of 0.002, 2 and 4 mg/kg, administered 4 times on alternate days (D1, D3, D5, D7). A two-way ANOVA for repeated measures revealed a significant effect for treatment (F_3,26_ = 8,863; p = 0,0003), time (F_4,12_ = 29,54; p < 0,0001) and treatment × time (F_12,104_ = 4,238; p < 0,0001); Tukey-Kramer post hoc test: PTX 0.002 (##) P < 0.005; PTX 2 (°) P < 0.05 and (°°) P < 0.005; PTX 4 (**) P < 0.005 vs CTRL; Inter-dose comparisons: D2 PTX 0.002 vs PTX 2 and PTX 4 (S) p < 0.05; D7 PTX 0.002 vs PTX 4 (S) p < 0.05. (CTRL N = 9; PTX 0.002 N = 6; PTX 2 N = 8; PTX 4 N = 7). In (C) example of footprints images derived from Vehicle (CTRL) or treated (PTX 4) animals at day 21 (D21). Toe spread (TS) and plantar length (PL) were measured, and sciatic static index (SSI) calculated as reported in methods. In SSI scale, values approximating −9,5 indicates normal functioning, while a value of −100 corresponds to complete functional loss. ANOVA for repeated measures revealed a significant effect for treatment (F_1,11_ = 9,89; p = 0,0093), time (F_1,1_ = 59,87; p < 0,0001) and treatment × time (F_1,11_ = 10,92; p = 0.007); Tukey-Kramer post hoc test: D21 CTRL vs PTX 4P < 0.005 (CTRL N = 6; PTX 4 N = 7). (D) Levels of circulating glucose (mg/dl) measured in naïve animals, control (CTRL – vehicle), paclitaxel (PTX – at the dose of 0.002, 2 and 4 mg/kg) – treated mice from the day after the first administration (day 2 – D2) and at D7, D14 and D21. A two-way ANOVA for repeated measures revealed a significant effect for treatment (F_4,40_ = 5,237; p = 0,0017), time (F_3,12_ = 4,498; p = 0,005) and treatment × time (F_12,120_ = 3,952; p < 0,0001); Tukey-Kramer post hoc test: D7) CTRL vs naïve (**) P < 0.005; naïve vs PTX 2 (*) p < 0.05; naïve vs PTX 4 (**) p < 0.005; D14) naïve vs PTX 4 (**) p < 0.005; D21) naïve vs PTX 4 (**) p < 0.005 (Naïve N = 12; CTRL N = 12; PTX 0.002 N = 6; PTX 2 N = 8; PTX 4 N = 7). (E) Levels of circulating triglycerides (mg/dl) measured in naïve animals, control (CTRL – vehicle), paclitaxel (PTX – at the dose of 0.002, 2 and 4 mg/kg) – treated mice from the day after the first administration (day 2 – D2) and at D7, D14 and D21. A two-way ANOVA for repeated measures revealed a significant effect for treatment (F_4,40_ = 7,44; p = 0,0001) and treatment × time (F_12,120_ = 3,61; p = 0,0001); Tukey-Kramer post hoc test: D2) CTRL vs naïve (**) P < 0.005; naïve vs PTX 0.002 (*) p < 0.05; naïve vs PTX 4 (**) p < 0.005; D14) naïve vs CTRL (**) p < 0.005; naïve vs PTX 0.002 (**) p < 0.005; D21) naïve vs CTRL (**) p < 0.005; naïve vs PTX 0.002 and PTX 2 (**) p < 0.005 (Naïve N = 12; CTRL N = 12; PTX 0.002 N = 6; PTX 2 N = 8; PTX 4 N = 7). (F) Correlation analyses between nociceptive thresholds and metabolic parameters. Significant negative correlations were found in PTX 0.002 group (aesthesiometer D2; Spearman r = −0.83, p = 0.042) and CTRL group (plantar D7; Spearman r = −0.82, p = 0.007), while a positive correlation was observed in PTX 2 group (plantar D7; Spearman r = 0.75, p = 0.031).

Our findings indicate that all doses caused neuropathic pain-like behaviour, manifesting as allodynia and/or hyperalgesia (Fig. 2A, B). Notably, mechanical allodynia was significantly observed in mice treated with 2 mg/kg on days 7 and 21, and in those treated with 4 mg/kg on days 14 and 21. There were no significant dose-dependent differences among the three treated groups, with lower-dose mice showing similar mechanical thresholds to those of higher-dose groups at all time points.

To evaluate paclitaxel-induced thermal hyperalgesia, we utilized the Plantar test. As shown in Fig. 2B, there was a decrease in withdrawal latency in treated mice from the day after the last injection (D7) to D21. The ultralow dose also produced a significant nociceptive response on days 7 and 14, while the 2 mg/kg dose consistently maintained thermal sensitization from D7 to D21. The higher dose (4 mg/kg) significantly induced hyperalgesia on D14. Importantly, in this test some inter-dose comparisons (Fig. 2B) reached significance (e.g., PTX 0.002 vs PTX 2 and PTX 4 at D2, and PTX 0.002 vs PTX 4 at D7), indicating partial dose-related variations in thermal hypersensitivity.

Vehicle-treated mice showed no significant alterations in mechanical or thermal thresholds across the experimental time course (baseline values vs time-points post treatment – paired *t*-test). Effect size analysis confirmed that significant differences corresponded to medium-to-strong effects in behavioural outcomes (Table S1).

To determine if peripheral nerves were also affected, we conducted a functional assessment of paclitaxel on PTX4- and CTRL-treated mice using walking track analysis. This test measures postural defects and alterations in plantar support during walking, providing insights into sciatic nerve function. For this, the Sciatic Static Index (SSI) was calculated as detailed in the Materials and Methods section, where a value of −9.5 indicates normal function, and −100 represents complete functional loss. This index is based on various measurements of hind paw footprints. In Fig. 2C, both experimental groups showed values close to the standard SSI at baseline. However, 21 days after starting PTX treatment, the chemotherapy-treated animals displayed footprint alterations, especially in plantar length (PL), indicating a significant sciatic functional defect.

### Effects of paclitaxel on metabolism

Since chemotherapeutic drugs can impact glucose and fat metabolism (Panis et al., 2017, Micheletti et al., 2021), these changes may be linked to both neuropathy (Vacca et al., 2023, Coccurello et al., 2018) and paclitaxel (PTX)-induced peripheral neurotoxicity (Bonomo et al., 2024). To investigate whether paclitaxel causes metabolic changes in mice and to explore any potential connections between metabolic dysfunction and neuropathic symptoms, in vivo metabolic measurements were conducted using blood samples. Specifically, glycemia and triglyceride levels were assessed.

As shown in Fig. 2, Fig. 2, fluctuations in glycemia and triglycerides coincide with the onset of neuropathy. Notably, mice treated with CTRL, PTX 0.002, and PTX 4 exhibited significant reductions in glycemia and increases in triglycerides as early as day 2 after starting chemotherapy, compared to naïve animals. These changes persisted on day 14 (approximately one week after the end of treatment) and day 21 (two weeks later) for the higher dose PTX 4 in glycemia, and for CTRL and PTX 0.002 on day 14, and CTRL, PTX 0.002, and PTX 2 on day 21 in triglyceride levels, when compared to naïve mice. Effect size analysis confirmed that significant differences corresponded to medium-to-strong effects, in metabolic outcomes (Table S1).

To explore whether metabolic alterations were directly associated with NeP onset and maintenance, correlation analyses were performed between nociceptive thresholds and metabolic parameters for each treatment group and time point (Fig. 2E). No significant correlations were observed for glycemia. Conversely, triglycerides showed three significant associations with pain sensitivity: in the PTX 0.002 group at day 2, higher triglyceride levels correlated with lower mechanical thresholds measured by aesthesiometer (Spearman r = –0.83, p = 0.042); in CTRL animals at day 7, triglyceride values were negatively correlated with plantar thresholds (Spearman r = –0.82, p = 0.007); and in PTX 2 animals at day 7, triglyceride levels showed a positive correlation with plantar thresholds (Spearman r = +0.75, p = 0.031).

It is known that the vehicle, Cremophor EL, is not an inert substance and can have significant biological effects, including hyperlipidaemia (Gelderblom et al., 2001).

### Paclitaxel-induced skin nerve endings sensitization

Henceforth, for molecular and histological analysis, we investigated paclitaxel doses (PTX 2 and PTX 4) commonly used to induce neuropathy in murine models, which also have translational relevance as anti-cancer mimicking therapeutic doses.

Since paclitaxel induce both mechanical allodynia and thermal hyperalgesia, we examined the expression and localization of transient receptor potential vanilloid 1 (TRPV1) and calcitonin gene-related peptide (CGRP) via immunohistochemical analysis in the skin of the mice hind paws.

TRPV1, a non-selective cation channel activated by capsaicin, heat, and acidic pH, mediates pain transduction and proinflammatory mediator release. It is expressed in peripheral nerve endings, keratinocytes, and skin structures involved in sensory perception. TRPV1 expression increases after nerve or skin injury, contributing to heat hyperalgesia.

**Fig. 3:**
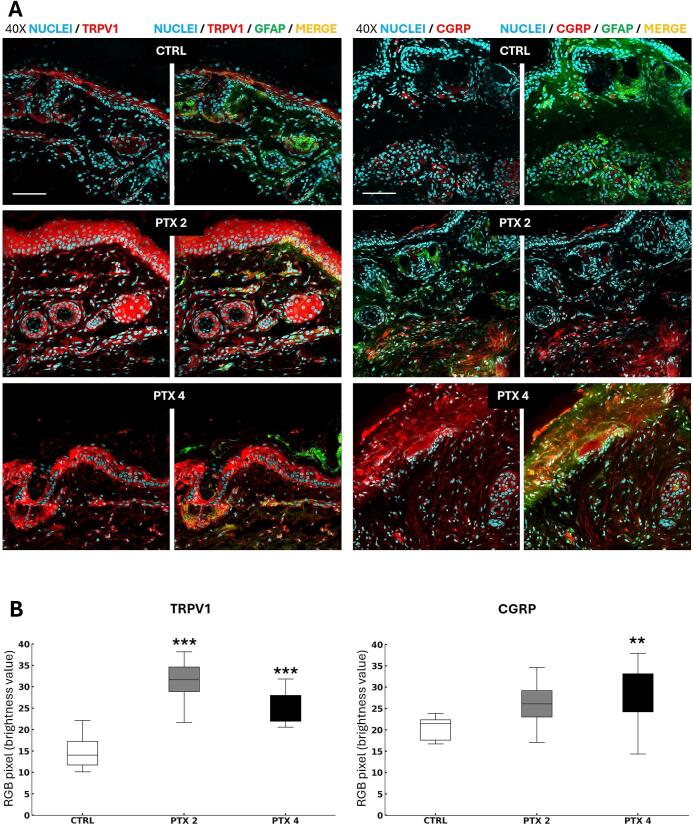
Paclitaxel affects nerve endings and skin receptors. (A) Representative 40x confocal images of double stained skin sections from the hind paws of vehicle-treated (CTRL) and paclitaxel-treated mice (PTX 2, PTX 4) 22 days after the start of the treatment. Scale Bar: 70 μm. (GFAP = green; TRPV1 or CGRP = red; Nuclei = cyan). Images revealed two main skin layers: the epidermis, composed by closely packed epithelial cells (keratinocytes), and the dermis, a dense connective tissue that houses blood vessels, hair follicles, sweat and sebaceous glands, free somatosensory nerve endings and tactile corpuscles (B) Expression of TRPV1 and CGRP (n = 3 animals per group, 3 images per animal) TRPV1: H_2_ = 17,802, p < 0,0001; CGRP: H_2_ = 8,89, p = 0,0117; Bonferroni-Dunn post-hoc test (***p < 0,0001, ** p < 0.005 vs CTRL;). (For interpretation of the references to colour in this figure legend, the reader is referred to the web version of this article.)

To assess whether paclitaxel upregulates TRPV1, we performed immunohistochemical analyses on the skin of the hind paws of treated and control mice. Fig. 3A shows representative images of skin sections (collected 22 days post-treatment) double-stained for glial fibrillary acidic protein (GFAP, a nerve fiber marker) and TRPV1. Fluorescence analysis revealed a significant increase in TRPV1 expression in both paclitaxel doses compared to vehicle-treated mice (Fig. 3B).

TRPV1 distribution in the skin also varied. Under control conditions, TRPV1 was sparsely distributed in keratinocytes (outer skin layer), sebaceous glands, nerve endings, blood vessels, and hair follicles, as observed in vehicle-treated mice. In PTX-treated mice, TRPV1 staining intensified, particularly in keratinocytes, sebaceous glands, blood vessels, and nerve endings in PTX2 samples, and in keratinocytes, hair follicles, associated sebaceous glands, and nerve endings in PTX4 samples.

Since CGRP, a neuropeptide involved in pain transmission and inflammation through immune cell activation and vasodilation, plays a crucial role, we performed IHC to investigate paclitaxel-induced changes in CGRP expression and localization.

Skin sections were stained with anti-GFAP and anti-CGRP antibodies (Fig. 3A). Notably, CGRP localization and CGRP-positive nerve fibres were more prominent in the epidermis of PTX4-treated animals. Immunofluorescence analysis revealed a significant increase in CGRP expression in the 4 mg/kg paclitaxel group compared to controls (Fig. 3B).

### Paclitaxel effect on peripheral nerve integrity

Demyelination is a common consequence of exposure to cytotoxic drugs or chemical insults (iatrogenic damage) (Hanewinckel et al., 2016, Zimmermann, 2001, Gierthmühlen and Baron, 2016, Cohen and Mao, 2014). In this study, we investigated paclitaxel's effects on Schwann cells (SCs) and peripheral myelin structure. To assess myelin integrity and protein levels, we performed immunostaining for P0 (the main peripheral myelin protein) and GFAP, alongside Western blot (WB) quantification of P0 and myelin basic protein (MBP) in sciatic nerves 22 days post-treatment. Given the potential involvement of autophagy and apoptosis in paclitaxel-induced nerve damage, we also analysed LC3 (an autophagy marker) and caspase 3 (an apoptosis marker).

**Fig. 4:**
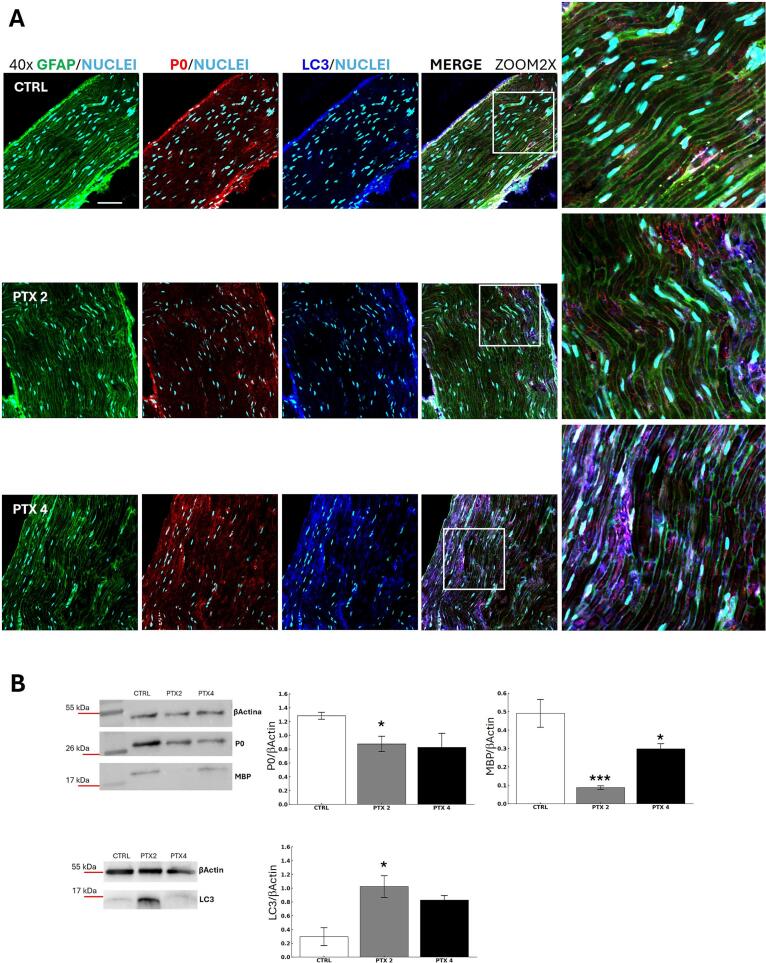
Detrimental effect of paclitaxel on Schwann cells. (A) Representative (40x and 2x zoom) confocal images of sciatic nerve sections stained for GFAP (green) and P0 (red) and LC3 (blue) from vehicle (CTRL) and paclitaxel-treated mice (PTX 2 and PTX 4) collected 22 days after the start of the treatment (Scale bar 70 μm). (B) Western blot quantification of MBP, P0 (PTX 2p = 0,0282 for P0; PTX 2p = 0,0193 and PTX 4p = 0,0004 for MBP) and LC3 (p = 0,0256 for CTRL vs PTX2) in sciatic nerves (CTRL n = 4; PTX2 n = 4; PTX4 n = 4; *p < 0,05, ***p < 0,0001 vs CTRL). (For interpretation of the references to colour in this figure legend, the reader is referred to the web version of this article.)

Confocal microscopy images (Fig. 4A) of sciatic nerve sections from vehicle-treated (CTRL) and paclitaxel-treated (PTX2, PTX4) animals stained for GFAP, P0, and LC3 showed normal nerve structure in controls, with intact SCs, myelin sheaths, and uniform P0 distribution. In contrast, paclitaxel-treated animals exhibited fragmented myelin sheaths and P0 aggregation, highlighting the neurotoxic effects of the drug. LC3 staining in SCs, typically marked by circular LC3 dots during autophagy, showed a general increase in LC3 expression without distinct dot formation, suggesting altered autophagy-related processes. WB analysis further confirmed a significant reduction in P0 and MBP levels in PTX2-treated sciatic nerves and reduced MBP in PTX4-treated nerves, consistent with demyelination (Fig. 4B).

To further explore autophagy involvement, WB analysis of LC3 was conducted to differentiate between total LC3-I and its lipidated form (LC3-II), which reflects autophagic activity. Results indicated a substantial increase in LC3-I levels in PTX2 and a slight increase in PTX4, but no detection of LC3-II, indicating no active autophagy (Fig. 4B).

Given the observed myelin degeneration and altered autophagy-related processes, we hypothesized that paclitaxel may also trigger apoptotic mechanisms. Caspase 3 analysis, however, revealed no significant differences in apoptosis across treatment groups (Supplementary Fig. 1), suggesting apoptosis may not play a major role in this context at the studied time points.

### Response of innate immunity to paclitaxel treatment

An assessment of innate immunity was conducted to determine if paclitaxel triggered an inflammatory response or initiated a degradation process.

**Fig. 5:**
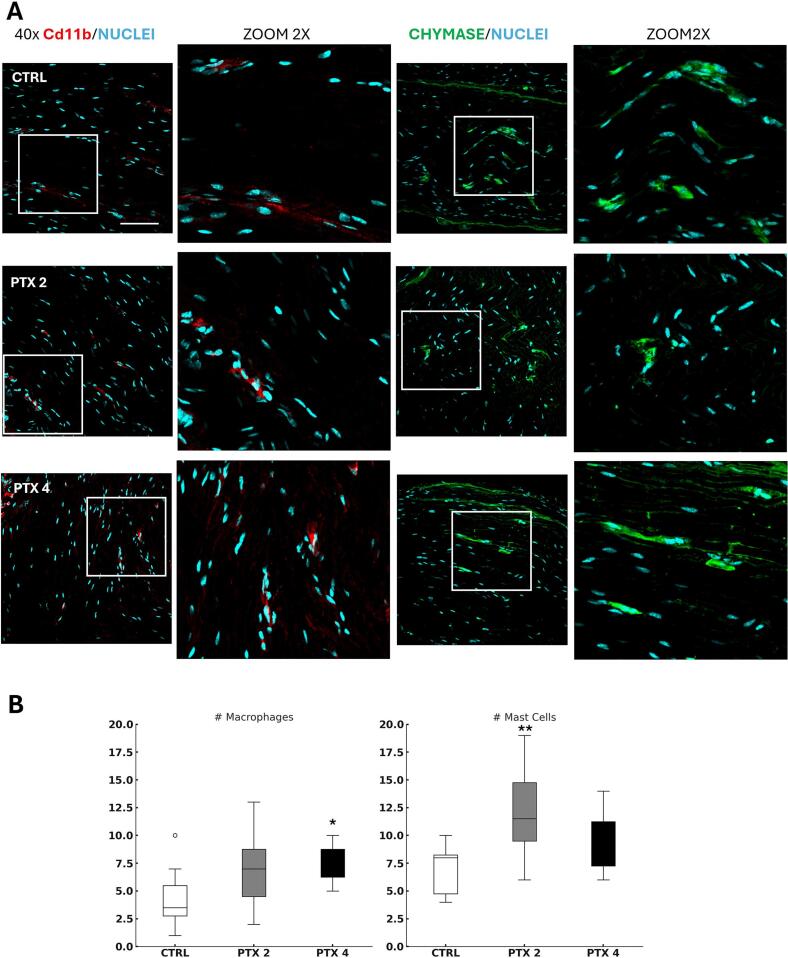
Macrophages and mast cells involvement in paclitaxel-induced nerve damage. (A) Representative 40x confocal images and 2x zooms from sciatic nerve sections of vehicle-treated (CTRL) and paclitaxel-treated mice (PTX 2, PTX 4). Macrophages are marked with anti-CD11b antibody (red) and mast cells with anti-chymase antibody (green); scale bar 70 μm. (B) Macrophages and Mast cells cell count (n = 3 animals per group, 3 images per animal) Cd11b: H_2_ = 5,777, p = 0.05; Chymase: H_2_ = 7,38, p = 0,0024; Bonferroni-Dunn post-hoc test (*p < 0,05, ** p < 0.005 vs CTRL). (For interpretation of the references to colour in this figure legend, the reader is referred to the web version of this article.)

Using immunofluorescence (Fig. 5A), we examined the number of macrophages (marked with Cd11b) and mast cells (marked with Chymase) in sciatic nerves from both control (vehicle-treated) and paclitaxel-treated animals (2 and 4 mg/kg), 22 days after the start of chemotherapy.

Cell counts revealed a significant increase in macrophages at the higher paclitaxel dose and a rise in mast cells at the lower dose (Fig. 5B).

## Discussion

Chemotherapy-induced peripheral neuropathy (CIPN) is a common and serious complication of chemotherapy, significantly impairing the quality of life for cancer patients and survivors. Despite ongoing research, the mechanisms underlying CIPN remain poorly understood. Over the past decade, numerous studies have attempted to identify the critical factors involved, but findings remain fragmented and inconclusive. A key challenge is the diversity of chemotherapeutic drugs, each of which induces unique toxic effects and involves distinct molecular pathways (Zajączkowska et al., 2019, Starobova and Vetter, 2017).

In this study, we investigated the dose-dependent effects of paclitaxel on peripheral nerve toxicity and neuropathic pain in mice. Behaviourally, all tested doses induced comparable levels of mechanical allodynia and thermal hyperalgesia, with no significant inter-dose differences, although the duration of symptoms was longer with higher doses (up to 21 days versus 14 days with the ultra-low dose). It is worth noting that in our study mechanical thresholds did not significantly differ between the 2 and 4 mg/kg groups. This finding is in line with previous reports showing that these two doses induce mechanical allodynia with a similar onset and magnitude (Toma et al., 2017), while only higher doses such as 8 mg/kg produce more robust and persistent effects. Moreover, recent data indicate that differences between dose regimens may emerge only at later stages, as strain-dependent pharmacokinetics and drug accumulation in dorsal root ganglia can shape the severity of neuropathy (Drabison et al., 2025). Thus, the apparent convergence of behavioral outcomes across 2 and 4 mg/kg may reflect assay ceiling effects and temporal dynamics, even though underlying molecular and histological alterations remain dose-dependent.

Of particular interest, the ultra-low dose of 0.002 mg/kg, intentionally chosen to probe sub-threshold effects, produced detectable responses in both behavioral and metabolic parameters. This highlights the potential vulnerability of peripheral nerves and systemic metabolism to even minimal chemotherapeutic exposure. Such findings have pharmacological relevance in terms of drug kinetics and dynamics, especially when considering cumulative low-dose regimens or unintended off-target effects in clinical settings. This suggests that even low doses of paclitaxel disrupt peripheral nerve function, but the magnitude and duration of this disruption increase with higher doses. Paclitaxel is known to cause peripheral nerve damage through mechanisms such as microtubule stabilization, leading to axonal degeneration, altered nerve conduction, and inflammatory responses (Staff et al., 2020, Kober et al., 2019, Bober et al., 2015). The extended duration of allodynia at higher doses may be attributed to more severe nerve damage, delayed repair, and sustained inflammation (La and Chung, 2017).

Thermal hyperalgesia followed a different pattern: it emerged earlier with the ultra-low dose (from day 7) but appeared later with higher doses (from day 14). This discrepancy suggests that different neurophysiological mechanisms may underlie paclitaxel-induced allodynia and hyperalgesia, or that different doses engage distinct pain pathways. It is possible that at ultra-low doses, thermal sensory fibers (C-fibers) are more susceptible to damage, leading to earlier hyperalgesia onset, while higher doses might cause more widespread neuropathic changes that delay the involvement of thermal pathways (Zajączkowska et al., 2019, Starobova and Vetter, 2017).

Both allodynia and hyperalgesia persisted for up to 21 days, suggesting that paclitaxel induces chronic pain states in this model. The continued presence of neuropathic symptoms after the cessation of paclitaxel injections mirrors clinical observations where patients undergoing chemotherapy often experience long-lasting neuropathic symptoms. The cumulative effects of paclitaxel, even at lower doses, can lead to lasting maladaptive changes in peripheral and central pain pathways, contributing to chronic pain development (Zajączkowska et al., 2019, Starobova and Vetter, 2017).

Peripheral nerve function can be affected by various mechanisms, including nociceptor sensitization and direct nerve damage. The Sciatic Static Index (SSI), which measures sciatic nerve function, revealed postural defects in mice treated with 4 mg/kg of paclitaxel, including altered paw support and toe abnormalities during walking, indicating mechanoreceptor involvement and damage to myelinated Aβ and Aδ fibers, typically not associated with nociceptive transmission (La and Chung, 2017).

Metabolic factors, such as hyperglycaemia and hypertriglyceridemia, also play a role in peripheral nerve toxicity. Schwann cell function is particularly vulnerable to these metabolic disturbances, which are common in conditions like diabetes and metabolic syndrome. Paclitaxel has been shown to disrupt lipid metabolism, altering enzymes, plasma proteins, and lipoproteins. However, there are conflicting reports on whether these changes represent upregulation or downregulation of metabolic pathways (Panis et al., 2017, Micheletti et al., xxxx, Coccurello et al., 2018, De Angelis et al., 2020). Recent studies have identified significant differences in plasma metabolites between paclitaxel-treated animals and controls, with elevated triglycerides, diglycerides, acylcarnitines, and ceramides confirming paclitaxel’s neurotoxic effects. Furthermore, the Triglyceride Glucose Index has been proposed as a predictor of paclitaxel-induced neuropathy (Bonomo et al., 2024, Gundogdu and Coban, 2022). Our results support these findings, as all doses of paclitaxel induced changes in glycemia and increased triglyceride levels. Interestingly, even the vehicle used (Cremophor) can affect triglyceride levels, highlighting its potential role in exacerbating peripheral neuropathy (Gelderblom et al., 2001). Metabolic variations in response to nerve injury have previously been described (Vacca et al., 2023, Coccurello et al., 2018) and are thought to reflect the increased energetic demands required to sustain repair processes and adaptive responses. In our study, we not only confirmed the presence of such changes but also correlated them with the emergence of neuropathic symptoms. Specifically, we found that TGs dynamics are differentially associated with neuropathic outcomes depending on both dose and time. The early correlation observed in the PTX 0.002 group suggests that even minimal exposure may be sufficient to trigger systemic metabolic changes linked to pain sensitization. Importantly, the negative association found in the CTRL group at day 7 should be interpreted in light of the vehicle used (Cremophor), which has been reported to increase triglyceride levels and alter lipid metabolism. This observation confirms that the vehicle itself may contribute to metabolic fluctuations and indirectly affect nociceptive thresholds. Finally, the positive correlation detected in PTX 2 animals indicates that lipid metabolism may contribute in a dose- and time-specific manner. Together, these data reinforce the hypothesis that dysregulated lipid metabolism is not only a systemic marker of stress but can actively influence the onset and trajectory of NeP.

Our histological analysis also showed dose-dependent involvement in peripheral nerve damage. Immunohistochemical and Western blot studies of the skin, which contains numerous mechanosensitive and nociceptive nerve endings, revealed significant paclitaxel-induced alterations. While much research has focused on the dorsal root ganglia and spinal cord, we found that chemotherapy-induced changes in somatosensory nerve endings are underexplored. The dermis and epidermis house free, unmyelinated C-fibers that act as nociceptors, and these fibers express receptors for various stimuli (Kennedy et al., 2005, Rice and Albrecht, 2008). Traditionally believed to function autonomously, recent research shows these nerve endings form complex structures with specialized nociceptive Schwann cells, crucial for pain perception (Abdo et al., 2019, Rinwa et al., 2021). Dysfunction in these structures likely underlies neuropathic symptoms such as allodynia and hyperalgesia (Hanewinckel et al., 2016, Zimmermann, 2001, Kennedy et al., 2005).

TRPV1, a non-selective cation channel involved in peripheral nociception and the release of pro-inflammatory mediators, is one such receptor. Its expression in paclitaxel-treated mice was significantly upregulated, correlating with the thermal hyperalgesia observed in behavioural tests (Marrone et al., 2017, Choi and Di Nardo, 2018). Additionally, CGRP-positive sensory fibers, which are involved in both pain perception and inflammatory responses, were more prominent in animals treated with the highest paclitaxel dose, indicating dose-related involvement of these fibers (Rice and Albrecht, 2008, Choi and Di Nardo, 2018, Russell et al., 2014).

Schwann cells, critical first responders to nerve injury, were also impacted by paclitaxel. These cells play a pivotal role in clearing myelin debris through autophagy, a process called myelinophagy, which begins shortly after nerve injury (Marinelli et al., 2014, Gomez-Sanchez et al., 2015). Myelin abnormalities were observed in nerve sections from paclitaxel-treated animals, with disrupted nerve fibers, myelin protein aggregates, and decreased expression of key myelin proteins such as P0 and MBP, indicating Schwann cell dysfunction (Rattanakrong et al., 2022, Imai et al., 2017). Elevated LC3 staining in sciatic nerve sections from paclitaxel-treated mice suggests altered autophagy-related processes, likely due to an overload of the process, but this requires further study (Kuma et al., 2007).

Despite the neurotoxic effects, there were no significant differences in apoptosis among treatment groups, suggesting that paclitaxel-induced nerve damage is primarily driven by neurotoxicity rather than widespread cell death. Moreover, immune cells such as macrophages and mast cells were elevated in paclitaxel-treated animals, with macrophages increasing at 4 mg/kg and mast cells at 2 mg/kg, indicating a dose-dependent immune response (Kiefer et al., 2001, Calvo et al., 2012, Mueller et al., 2001).

Our findings on sensory nerve sensitization and innate immune involvement are consistent with prior reports in paclitaxel neuropathy. In particular, Gao et al. (2016) showed that stabilizing mast cells with quercetin mitigates paclitaxel-induced mechanical and thermal hypersensitivity while suppressing PKCε-dependent TRPV1 activation in DRG and spinal cord, directly linking mast-cell degranulation to nociceptor sensitization (Gao et al., 2016). In parallel, Lucarini et al. (2023) demonstrated broad-spectrum neuroprotection in a paclitaxel model, with recovery of sensory nerve conduction and preservation of intraepidermal nerve fibers and neurofilament integrity, underscoring that tissue-level injury and small-fiber loss accompany behavioral allodynia (Lucarini et al., 2023). These converging data reinforce a dual-hit mechanism in our study, immune-driven TRPV1 sensitization together with peripheral nerve structural vulnerability, and provide a mechanistic framework for our observation that behavioral thresholds can converge across doses even when histological and molecular alterations remain dose-responsive.

This study significantly advances our understanding of CIPN. By elucidating the dose-dependent effects of paclitaxel on nerve damage and identifying key molecular players, such as TRPV1, CGRP, and Schwann cell dysfunction, our findings provide a more detailed map of the pathogenic mechanisms underlying CIPN. This knowledge is crucial for developing more targeted strategies to prevent or mitigate the painful consequences of chemotherapy, improving the quality of life for cancer patients and survivors.

The insights gained from this study open several avenues for future research and clinical applications. Targeting specific pathways involved in paclitaxel-induced nerve damage, such as TRPV1 or Schwann cell repair mechanisms, could offer new therapeutic approaches for managing CIPN. Additionally, the potential role of metabolic disturbances, such as hypertriglyceridemia (partially due to paclitaxel vehicle), in exacerbating nerve toxicity suggests that metabolic modulation could be explored as a complementary treatment strategy as well as other less toxic vehicles can be investigated. Future investigations should focus on the development of biomarkers to predict CIPN susceptibility and the exploration of neuroprotective agents that could be co-administered with paclitaxel to prevent or reduce its neurotoxic effects.

In conclusion, this study provides a comprehensive framework for understanding the complex mechanisms of paclitaxel-induced peripheral neuropathy, with significant implications for both basic research and clinical practice.

## Limitations and future directions

The present study provides new insight into the peripheral and metabolic alterations underlying paclitaxel-induced neuropathy, but it also opens important questions that warrant further investigation. While behavioural testing confirmed the development of mechanical allodynia and thermal hyperalgesia across doses, additional paradigms such as cold allodynia, spontaneous pain, and motor impairment could extend our understanding of the multidimensional nature of chemotherapy-induced neuropathy and better reproduce the clinical scenario.

At the molecular level, our findings revealed an increase in LC3 expression, pointing to an involvement of autophagy-related pathways. Although the exact nature of this alteration (induction vs. blockade of autophagic flux) remains to be determined, this observation highlights autophagy as a promising mechanism to explore in future studies, particularly in relation to Schwann cell homeostasis and myelin integrity. Similarly, the increased presence of macrophages and mast cells suggests an immune contribution to the pathology. Phenotypic characterization of these cells (e.g., pro-inflammatory vs. pro-regenerative) will be crucial to clarify their dual role in injury progression and repair.

Finally, our choice of Cremophor EL as a vehicle was guided by its clinical relevance as part of the original Taxol® formulation. The observation that metabolic alterations also occurred in vehicle-treated animals underlines the importance of considering the solvent’s contribution and points to the need for comparative studies with Cremophor-free paclitaxel preparations.

In summary, this work sets the stage for future research aimed at dissecting the interplay between metabolic imbalance, immune activation, and glial cell responses in chemotherapy-induced neuropathy. Addressing these directions will be essential to delineate the mechanisms that drive disease evolution and to identify potential therapeutic targets.

## CRediT authorship contribution statement

**Maria Maiarù:** Writing – review & editing, Supervision, Methodology, Funding acquisition, Formal analysis, Conceptualization. **Andrea Petrini:** Writing – original draft, Investigation. **Federica De Angelis:** Writing – review & editing, Methodology, Investigation. **Francesca Nazio:** Writing – review & editing, Formal analysis, Conceptualization. **Sara Marinelli:** Writing – original draft, Supervision, Methodology, Formal analysis, Data curation, Conceptualization.

## Declaration of competing interest

The authors declare that they have no known competing financial interests or personal relationships that could have appeared to influence the work reported in this paper.

## Data Availability

Data will be made available on request.
