# Supplementary material for: Unveiling the peripheral nerve hallmarks of chemotherapy-induced neuropathy: insights from paclitaxel treatment in a murine model
Source: Neurobiol Pain. 2025 Oct 8;18:100200. doi: 10.1016/j.ynpai.2025.100200 (PMC12549393; doi:10.1016/j.ynpai.2025.100200)
Supplement: Supplementary Data 1 [file mmc1.docx]

**Supplementary materials**

**Unveiling the Peripheral Nerve Hallmarks of Chemotherapy-Induced Neuropathy: Insights from Paclitaxel Treatment in a Murine Model**

**Authors:** Maria Maiarù^1^, Andrea Petrini^2^, Federica De Angelis^3^, Francesca Nazio^4^, Sara Marinelli^3^

**Corresponding author:** Sara Marinelli, PhD – [sara.marinelli@cnr.it](mailto:sara.marinelli@cnr.it)

**Affiliations:**

**^1^** Department of Pharmacology, School of Pharmacy, University of Reading, Reading RG6 6UB, United Kingdom

^2^ Sapienza University of Rome, Faculty of Mathematical, Physical and Natural Sciences, Department of Biology and Biotechnology “Charles Darwin” Neurobiology Degree, Rome, Italy

^3^ National Council of Research, Institute of Biochemistry and Cell Biology (IBBC), Monterotondo (RM), Italy

^4^ Department of Biology, University of Rome Tor Vergata, Rome, 00133, Italy

**Figure S1**

**
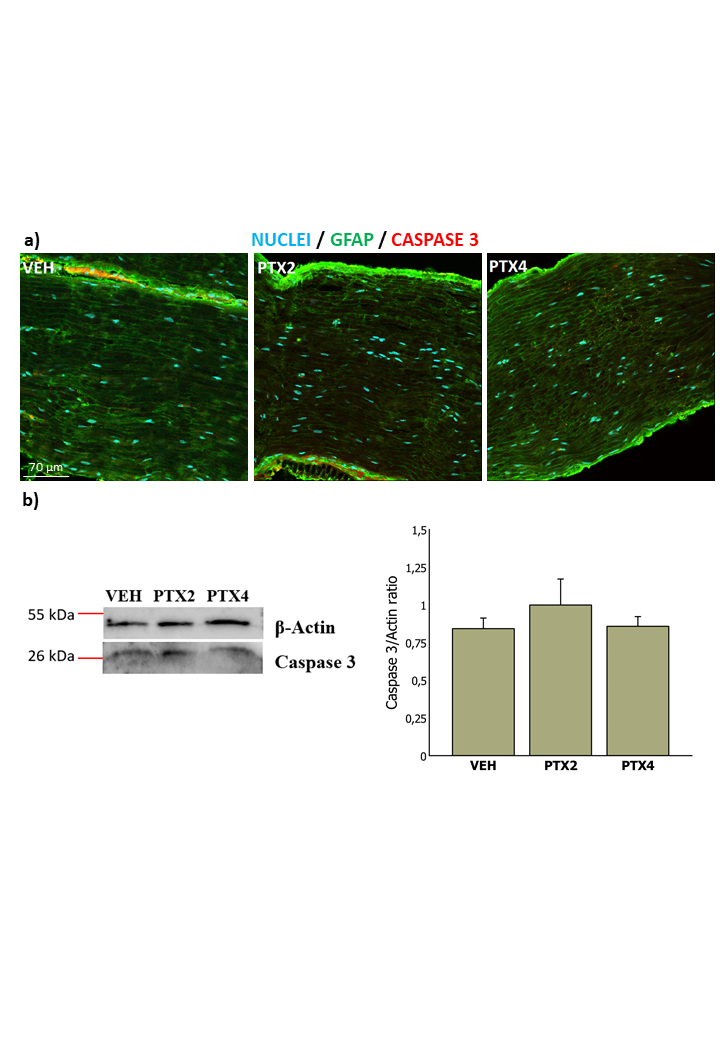
**

Figure S1. (a) Representative 40x confocal images of sciatic nerve sections double stained with anti-GFAP (green) and anti-caspase 3 (red) antibodies from vehicle-treated (VEH) and paclitaxel-treated mice (PTX2 and PTX4) collected 22 days after the start of the treatment. (b) Western blot quantification of caspase 3 expression in sciatic nerves from vehicle-treated and paclitaxel-treated animals (n=4).

**Table S1 – Effect sizes and Confident Interval for significant results**

| **Test / Outcome** | **Comparison (Day)** | **Effect size (Cohen’s d)** | **95% CI** | **Significance** |
| --- | --- | --- | --- | --- |
| Aesthesiometer – Mechanical Allodynia | CTRL vs PTX 2 (D7) | 1.05 | [0.30 – 1.72] | ** |
| Plantar Test – Thermal Hyperalgesia | CTRL vs PTX 0.002 (D7) | 0.92 | [0.20 – 1.60] | ** |
| Plantar Test – Thermal Hyperalgesia | CTRL vs PTX 4 (D14) | 1.20 | [0.50 – 1.85] | *** |
| Metabolic – Triglycerides | CTRL vs PTX 0.002 (D2) | 0.88 | [0.10 – 1.55] | * |
| Metabolic – Glycemia | CTRL vs PTX 4 (D21) | 1.15 | [0.45 – 1.80] | ** |

**Table S1.** Effect sizes (Cohen’s *d*) and 95% confidence intervals (CI) for significant results in behavioural and metabolic outcomes. Reported comparisons include treatment groups versus control (CTRL) at specific time points. Symbols indicate statistical significance as reported in the main figures (*p < 0.05; **p < 0.01; ***p < 0.001).
